# Nutritional Assessments by Bioimpedance Technique in Dialysis Patients

**DOI:** 10.3390/nu16010015

**Published:** 2023-12-20

**Authors:** Jack Kit-Chung Ng, Sam Lik-Fung Lau, Gordon Chun-Kau Chan, Na Tian, Philip Kam-Tao Li

**Affiliations:** 1 Carol and Richard Yu Peritoneal Dialysis Research Centre, Department of Medicine & Therapeutics, Prince of Wales Hospital, The Chinese University of Hong Kong, Hong Kong 999077, China; nkc426@ha.org.hk (J.K.-C.N.); llf790@ha.org.hk (S.L.-F.L.); cck295@ha.org.hk (G.C.-K.C.); 2 Department of Nephrology, General Hospital of Ningxia Medical University, Yinchuan 750004, China; tianna@nxmu.edu.cn

**Keywords:** nutrition, bioimpedance, end-stage kidney disease, dialysis, protein-energy wasting

## Abstract

Bioelectrical impedance analysis (BIA) has been extensively applied in nutritional assessments on the general population, and it is recommended in establishing the diagnosis of malnutrition and sarcopenia. The bioimpedance technique has become a promising modality through which to measure the whole-body composition in dialysis patients, where the presence of subclinical volume overload and sarcopenic obesity may be overlooked by assessing body weight alone. In the past two decades, bioimpedance devices have evolved from applying a single frequency to a range of frequencies (bioimpedance spectroscopy, BIS), in which the latter is incorporated with a three-compartment model that allows for the simultaneous measurement of the volume of overhydration, adipose tissue mass (ATM), and lean tissue mass (LTM). However, clinicians should be aware of common potential limitations, such as the adoption of population-specific prediction equations in some BIA devices. Inherent prediction error does exist in the bioimpedance technique, but the extent to which this error becomes clinically significant remains to be determined. Importantly, reduction in LTM has been associated with increased risk of frailty, hospitalization, and mortality in dialysis patients, whereas the prognostic value of ATM remains debatable. Further studies are needed to determine whether modifications of bioimpedance-derived body composition parameters through nutrition intervention can result in clinical benefits.

## 1. Introduction

Approximately 700 million people worldwide are estimated to be undergoing different stages of chronic kidney disease (CKD), thus resulting in a global prevalence of 9.1% [1]. Malnutrition is a well-recognized complication among patients with end-stage kidney disease (ESKD), and it has been consistently shown to predict increased risks of cardiovascular events and mortality together with prolonged hospital stays [2,3,4,5].

Given the strong association between malnutrition, wasting, and inflammation, the International Society of Renal Nutrition and Metabolism (ISRNM) proposed the term ‘protein-energy wasting’ (PEW), which refers to a state of decreased body protein stores and energy reserves [6]. It should be emphasized that PEW is not entirely explained by inadequate nutrient intake, but it is a hypercatabolic state driven by sustained inflammation and hormonal disturbances. The diagnosis of PEW requires the fulfilment of at least three out of four criteria, including abnormal serum biochemistry (low albumin or prealbumin), decreases in body mass (a low body mass index (BMI) or a total body fat of <10%), reductions in muscle mass, and low protein or energy intake [6]. 

The accurate measurement of body compositions is crucial since it constitutes two domains when establishing a diagnosis of PEW. However, the position paper by ISRNM did not recommend a preferred methodology, which may potentially create confusion among clinicians. A meta-analysis comprising 16,434 prevalent dialysis patients from 34 countries reported that the interquartile range for the prevalence of PEW was 28–54%, with substantial heterogeneity found among studies [7]. This may, at least partly, be attributed to the different diagnostic criteria applied between studies, as well as the variability in assessment tools [7]. Although dual X-ray absorptiometry (DEXA) is considered the gold standard for measuring the body composition in CKD patients, its generalizability in daily clinical practice is limited by cost and availability [6]. Anthropometric parameters, such as BMI, are easy to use but do not provide information on different body compartments. A low BMI (e.g., <23 kg/m^2^ according to the criteria proposed by ISRNM [6]) does not necessarily indicate wasting, especially in Asians. Conversely, an increase in BMI does not differentiate between a gain in muscle or adipose mass in CKD patients, in whom sarcopenic obesity is not uncommon and associated with adverse outcomes [8]. Composite nutritional indices, including subjective global assessment (SGA) and the malnutrition–inflammation score (MIS), have been increasingly used in the assessment of nutritional status and/or to support the diagnosis of PEW in dialysis patients [9,10]. However, several versions (five-point or seven-point scales) of SGA were available after modifications from the original one by Detsky et al. [11]; in addition, clinicians have adopted different cut-offs to define malnutrition or PEW [11]. Moreover, the subjective consideration of aggregate data to determine the final score of SGA could possibly increase inter-observer variability [12]. This is further complicated by the lack of consensus on the diagnostic cut-off of SGA or MIS to define PEW [6,7].

The bioimpedance technique has emerged as a promising alternative through which to measure whole body compositions among nephrologists in the past two decades [13,14]. In the general population, bioelectrical impedance analysis (BIA) has been widely applied to assess and monitor nutrition in patients with human immunodeficiency virus infection [15], liver cirrhosis [16], and malignancy [17]. The importance of quantifying muscle mass was further highlighted in the latest consensus statement by the Global Leadership Initiative on Malnutrition (GLIM), which separated ‘reduction in muscle mass’ from ‘low BMI’ as a distinct phenotypic criterion to diagnose malnutrition [18]. Notably, BIA is one of the validated methods to assess muscle mass as recommended by GLIM [18]. In ESKD patients, BIA has an additional advantage in objectively measuring the degree of volume overload, which has been shown to predict patient and technique survival [19,20,21]. Despite the evolution of bioimpedance technology, there remains concern on the use of BIA in the nutrition management of ESKD patients. In this narrative review, we aim to discuss the basic principles of bioimpedance, its application in measuring body composition and its associated limitations, and to explore the role of bioimpedance on nutrition management in dialysis patients.

## 2. Principles and the Validation of Bioimpedance in ESKD

### 2.1. Basic Technological Principles of the Bioimpedance Technique

Since the publication of the seminal paper by Lukaski and colleagues in 1985, bioimpedance techniques have gained considerable popularity among physicians because they enable simple, non-invasive measurements of body composition at bedside [22]. The methodological details were extensively discussed in previous reviews [23,24]. Essentially, bioimpedance devices apply low amplitude alternating currents to the body via electrodes attached to the surface of limbs (most commonly those in a tetrapolar arrangement, i.e., one hand and one foot). The ‘impedance’ of a conductor (human body) is a function of resistance and reactance. Resistance refers to the opposition of the flow of the current by body fluids, and it is inversely proportional to the total body water (TBW), whereas reactance is a measure of cell capacitance and is directly related to the body cell mass [13]. The arc tangent ratio of reactance to resistance is denoted by the phase angle (PA), which represents a phase shift of alternating currents due to the temporary storage of charges by cell membranes [24]. By fitting bioimpedance data into device-specific mathematical algorithms, the volume of body compartments (extracellular and intracellular water (ECW and ICW)) can be calculated.

Through technological innovations, current bioimpedance devices have evolved from applying a single frequency (SF-BIA), multiple fixed frequencies (multi-frequency BIA and MF-BIA), to over a range of frequencies (bioimpedance spectroscopy, BIS). The advantage of passing different frequencies of current (in contrast to a single fixed 50 kHz in SF-BIA) by MF-BIA and BIS allows for a better appreciation of body compartment volumes, as low-frequency currents mainly pass through ECW and high-frequency ones transverse cell membranes (thus TBW can be quantified [22]). Measurements can also be performed as ‘whole body’ (wrist-to-ankle) or ‘segmental’ (limbs or trunk).

There are a few key differences between BIA (SF-BIA or MF-BIA) and BIS, which lie in the categorization of body compartments (Table 1). BIA assumes a two-compartment (2C) model, which consists of fat mass (FM) and fat-free mass (FFM), and is derived from TBW under the assumption that FFM is constantly hydrated at 73.2% [23,24]. On the other hand, BIS is based on a three-compartment (3C) model, which consists of normally hydrated lean tissue mass (LTM), normally hydrated adipose tissue mass (ATM), and the volume of overhydration (OH) (the difference between estimated and measured ECW) [25]. Another major difference is the expression of BIA data (Table 1). The output from SF-BIA and MF-BIA typically includes vector plots and PAs. While the former may be useful in visualizing the longitudinal changes of body composition, it does not guide nephrologists on the magnitude of volume or adiposity excess [13]. Although it was evident that the PA was inversely associated with mortality in ESKD patients from a recent meta-analysis [26], there was no consensus on the normal range of PA out of which the dialysis patients were considered to be malnourished. In contrast, BIS-derived LTM and ATM are expressed in kilograms, which can also be converted to the lean tissue index (LTI) and fat tissue index (FTI), respectively, by dividing by height in meters squared. One of the most commonly used BISs, the Body Composition Monitor (Fresenius Medical Care, Bad Homburg, Germany), may have an additional advantage because it provides a reference range (10th and 90th percentile) of the OH, LTI, and FTI derived from 1247 healthy Caucasians controls [27].

### 2.2. Limitations and Challenges

It is important to recognize the limitations of BIA. First, SF-BIA and MF-BIA devices apply prediction equations that are derived from age-, gender-, and ethnicity-specific reference populations. As a result, biases may occur if these equations are applied to subjects that have different demographics from the reference population [28]. For example, BIA equations that are specifically generated from a sample of elderly subjects could predict appendicular skeletal muscles mass better than equations derived from a sample population of all ages [28]. Some of the commonly employed prediction equations and reference values are summarized in Table 2 and Table S1, respectively [29,30,31,32,33]. Second, the original whole-body SF-BIA assumes that the body is a single cylinder with uniform conductivity. However, this model is likely to be physiologically inappropriate because the trunk contributes disproportionally greater conductor volume than resistance when compared to four limbs [24]. In addition, the assumption of a constant ratio between ECW to ICW may be violated in altered body compositions such as obesity and volume overload [24]. It is also questionable whether the use of a single frequency in SF-BIA (or a few fixed frequencies in MF-BIA) could adequately differentiate between ICW and ECW. Third, the 2C model of BIA uses a fixed hydration constant of 0.732 to calculate FFM, which does not differentiate between excessive ECW and lean tissue water. This possibly results in an overestimation of FFM, especially in patients with fluid retention and obesity [28,34].

There are some potential solutions to these limitations. Clinicians may choose to use raw bioimpedance data directly as a nutrition assessment to minimize the error introduced by inappropriate prediction equations. For example, PA is directly derived from resistance and reactance, which are typically measured at 50 kHz. PA was shown to be an independent predictor of clinical outcomes in dialysis patients (see Section 3 below). The major pitfall of this approach is the lack of consensus on the cut-off that could best identify patients with malnutrition (or poor clinical outcomes) [24]. Similarly, bioelectrical impedance vector analysis (BIVA) provides a graphical expression by plotting both resistance and reactance (via SF-BIA) normalized to height [35]. This generates a vector plot that can then be compared with the reference values from a healthy population. But both PA and BIVA do not quantify individual body compartments that are essential for the diagnosis of PEW.

MF-BIA and BIS are considered to be superior to SF-BIA because they use multiple, or a range of, frequencies that better distinguish ECW and ICW. Segmental measurements with MF-BIA may produce more accurate body estimates than the whole-body measurements of SF-BIA because the former separates the body into five cylinders, whereas impedances are measured separately. Furthermore, BIS may have a theoretical advantage over BIA because it does not rely on population-specific prediction equations but instead determines body composition under a wide spectrum of frequencies by fitting impedance data into the Cole model [24]. This algorithm applies different resistivity constants in assessing each body compartments, which may be potentially beneficial in patients with altered body composition because it does not assume a constant ECW to ICW ratio as in SF-BIA. Moreover, BIS measures OH (defined as the difference between predicted and measured ECW) by assuming ‘normally hydrated’ LTM and ATM [25]. Specifically, this 3C model recognizes the dissimilar hydration status of LTM and ATM, which contrasts with the aggregate of excessive fluid, lean, and adipose tissue water in the FFM of the 2C model.

### 2.3. Practical Considerations during Bioimpedance Measurement

The accuracy of bioimpedance measurement may be undermined by non-standardized measurement procedures. Table 3 summarizes the key domains that should be standardized during bioimpedance measurements. Patients with cardiac pacemakers or implantable defibrillators are contraindicated to bioimpedance studies. Although it has been suggested that the bioimpedance measured on the contralateral side of metallic implants is acceptable [34], the precision of the measurement is understandably affected by the length of the implant and the conductivity of the metal [36]. Since the impact of metallic implants on impedance values may not be easily predicted (though it is believed to be small [36]), we caution against the application of bioimpedance in this situation. The instruments used during measurement may also affect reliability. For example, proximity of electrodes (placed within 5 cm) may lead to magnetic interference and thus should be avoided [28]. It was also observed that impedance values are inversely correlated with skin temperature [37]. Thus, it is not advisable to perform bioimpedance in febrile subjects with possible cutaneous vasodilation. Moreover, alcohol is commonly applied on the skin surface to remove secretions and improve the conductivity of electrodes. However, it is not certain such practices are consistent across dialysis centers, in which the procedures could be carried out by untrained dialysis assistants or nurses, which could potentially increase inter-observer variability. Food intake and exercise before bioimpedance application has been shown to reduce impedance values by various extents and should be avoided [38]. It is also important to note that several other factors may also alter influence resistance and/or reactance, including pregnancy, infection, tumor, endocrine disorder (acromegaly), extremes of BMI (<16 kg/m^2^ or >34 kg/m^2^), or ascites [34].

In fact, the dialysis procedure itself may also introduce errors in bioimpedance measurement. There has been a continuous debate on the effect of indwelling peritoneal dialysate on the BIA-derived parameters in patients on peritoneal dialysis (PD). Earlier studies have showed that MF-BIA (InBody 720, Biospace, Republic of Korea) measurements with PD fluid in situ significantly overestimate FFM, with conflicting effects on FM [39,40]. Subsequent studies using BIS (Body Composition Monitor, Fresenius Medical Care, Bad Homburg, Germany) did not detect any difference in the LTM and ATM before and after the drainage of PD fluid [41,42], albeit a small but significant increase in OH was observed when the PD fluid was instilled into the abdomen [41]. It was believed that electric currents travel through pathways that have the least resistance; as such, the trunk contributes only a minority of the impedance compared with limbs, which contain a higher density of muscles (and more water) [13]. Interestingly, a recent study that compared BIS-derived parameters in 31 liver cirrhosis patients before and after abdominal paracentesis revealed that the OH and LTM were not modified after drainage of ascitic fluid [43]. Nevertheless, an extrapolation of these results to PD patients may not be easy because the mean drainage volume of ascites is 7.8 L, which is nearly four times greater than the conventional volume of PD fluid. On the other hand, the procedures of measurement were not uniform in the aforementioned studies, with one of them being performed in a standing position [40], one in a supine position [41], and a position that was not mentioned in the rest [39,42]. The standing position was associated with a transient decrease in impedance due to the pooling of blood into lower extremities [28], thereby potentially leading to erroneous results. Similarly, only one of the studies specified that the BIS measurements were repeated with the same electrodes with a 10 min rest after PD fluid was drained out [41]. More importantly, the observed differences in the FFM, although statistically significant, were generally less than 5% [39,40], but this might not translate into meaningful clinical significance. Therefore, although it is ideal to measure the bioimpedance in PD patients after the drainage of PD fluid, it is our opinion that a ‘full abdomen’ is acceptable in order to streamline logistics in daily clinical practice, provided that the standardized measurement procedures (including proper skin preparation, electrode placement, patient position, and specified device model) are implemented by trained personnel [44,45].

The optimal timing to perform bioimpedance in patients on hemodialysis (HD) was studied by comparing the body composition parameters before and after a HD session. Panorchan et al. measured segmental MF-BIA (InBody 720, Derwent Health Care, Seoul, Republic of Korea) in 676 European HD patients in a midweek dialysis session, and they revealed a significant decrease in the FFM with a reciprocal increase in the FM after dialysis [46]. Change in the ECW during HD was positively correlated with a change in the skeletal muscle mass, and it was inversely correlated with a change in the percentage of body fat, thus suggesting the measurement of these nutrition parameters may be confounded by rapid fluid shifts during HD [46]. The same group of investigators conducted a similar study with a 3C-model BIS (BodyStat multiscan 5000, BodyStat, Isle of Man, Doulgas, UK) on pre- and post-dialysis measurements of 48 HD patients [47]. The results were largely consistent with their previous studies with BIA. Therefore, the investigators proposed that bioimpedance measurements should preferably be performed post-dialysis when patients are close to their target weight [46,47]. We suggested measuring bioimpedance at least 60 min after the end of a HD session to allow equilibrium to be reached between the body compartments.

### 2.4. Validation Studies of the Bioimpedance Technique in ESKD Patients

The enthusiasm for the clinical application of bioimpedance in the past two decades has driven numerous research efforts that have aimed to validate it against a ‘reference method’, of which DEXA is the most commonly regarded as the gold standard.

Most of the studies demonstrated strong correlations (r > 0.7) between the parameters derived from bioimpedance techniques and those from DEXA in both PD and HD patients [48,49,50,51,52,53]. Konings et al. were one of the first who compared the body composition parameters measured by MF-BIA and DEXA in 40 Caucasian PD patients [48]. They did not find any systematic bias in the estimation of the FFM and FM between MF-BIA and DEXA, respectively, but the limits of agreement between the methods could be as wide as ±8 kg [48]. Among 72 European PD patients, the mean difference in the estimated FM and FFM between DEXA and BIS was 0.9 ± 5.7 kg (95% confidence interval [CI] = −10.5–12.3) and −0.3 ± 5.6 kg (95% CI = −11.8–10.8), respectively [49]; however, the large standard deviation may otherwise suggest wide individual variability. These findings were confirmed in another study consisting of 50 PD patients, which again showed a good agreement in the FFM between BIS and DEXA. But BIS significantly overestimated FM by an average of 2.5 kg compared with DEXA, which became more pronounced in obese individuals [50].

In a single-center cross-sectional study of 60 HD patients, the performance of MF-BIA (Quadiscam 4000, Bodystat, UK) and the skinfold thickness sum before and after HD were compared using air displacement plethysmography as the standard method [54]. MF-BIA was found to underestimate FM and overestimate FFM when compared with air displacement plethysmography, while the discrepancy in the latter became insignificant after HD [54]. This again reinforced the importance of measuring bioimpedance post-dialysis. Skinfold thickness sum, on the other hand, showed a similar estimation of FM and FFM compared to the standard method. But it should be noted that the mean BMI of this cohort was 22 kg/m^2^, and it remained uncertain whether the accuracy of skinfold thickness can be maintained in obese patients (whose bony prominence is obscured). Other studies using DEXA as reference method reported no significant difference compared with skinfold thickness and BIA/BIS, albeit with a variable limit of agreement [53,55]. Notably, Bross et al. showed that SF-BIA with the Kushur equation had the lowest prediction error in estimating the percentage of body fat in 118 HD patients, thereby indicating the performance between the prediction equations were not identical [55].

The latest Kidney Disease Outcomes Quality Initiative (KDOQI) guideline cautioned about the impact of hypervolemia on the accuracy of nutritional assessments when conducted by the bioimpedance technique [56]. This error may not be surprising in 2C-based BIA because excessive ECW and lean tissue were included in the same compartment. As such, the FFM may be easily overestimated in patients who have volume overload. A previous study on a small cohort of PD patients supported this notion by showing a significant correlation between the FFM (by MF-BIA) and the left ventricular end-diastolic diameter (LVEDD), which was used as a surrogate marker for volume status [48]. However, LVEDD may be confounded by pre-existing structural heart diseases. A recent study explored the relation between BIS-derived nutrition indexes and echocardiographic parameters, including the ratio of peak early mitral inflow velocity to mitral annulus early diastolic velocity (E/e’), as well as to the left atrial volume index (LAVi), in 101 incident Chinese PD patients [57]. These two parameters were more reliable in assessing LV filling pressure than LVEDD [58], and thus were selected to reflect intravascular volume. Both the LTI and FTI were not associated with E/e’ or LAVi, thus suggesting the bioimpedance-derived nutrition indexes were not biased by volume overload [57]. When using BIS-derived hydration parameters, the difference in the FFM estimated by DEXA versus the bioimpedance technique were shown to correlate with the OH in some studies [49,53] but not others [50], and this effect was apparently more obvious in PD patents [53]. It is, however, crucial to realize that FFM measurement by DEXA itself is also influenced by hydration status [59]. The systematic bias between DEXA and the more accurate four-compartment model (including isotope dilution and hydrodensitometry) has challenged the reliability of DEXA as the gold standard [60].

How do clinicians reconcile the widespread utility and impreciseness of the bioimpedance technique in daily practice? Most of the studies showed that bioimpedance had a satisfactory agreement with reference methods at the population level but not the individual level. More importantly, the most commonly used reference method (DEXA) has its own limitations, which reinforces the fact that no single measurement method is free of error [14]. In contrast to ‘statistical significance’, clinicians may be more interested in their ‘clinical significance’. The absolute difference between measurements by the bioimpedance and reference methods were usually small, but whether they were clinically acceptable requires further outcome studies. The controversy on hypervolemia on the precision of bioimpedance is likely to continue, although BIS might be less biased by it considering a physiological 3C model. While it seemed logical to perform nutritional assessments with the bioimpedance technique after a normalization of volume status, it was not always feasible given the high prevalence of overhydration in dialysis patients, which persisted over time [19,20]. Interestingly, by defining a margin of error as exceeding ±2 kg (the difference between DEXA and BIS), a prospective study reported that a cut-off of ≥0.725 of the ratio of ECW to ICW (E/I ratio) may signify an inadequate error tolerance in FFM estimation (sensitivity: 80%; specificity: 30%) [61]. Since this study included a case mix of young pre-dialysis and dialysis patients, further studies to validate this cut-off in dialysis patients are required. However, it did raise a question about the level of hypervolemia that would render bioimpedance-derived nutritional indexes unreliable.

## 3. Association of Bioimpedance-Derived Nutritional Parameters and Clinical Outcomes

Owing to the non-invasiveness, portable, and highly reproducible nature of the bioimpedance technique, it has been extensively used in dialysis patients to assess and monitor volume status. Previous studies showed that baseline and persistent hypervolemia are both independent predictors of inferior patients and technique survival [19,20,21]. Here, we reviewed the published evidence on the association between bioimpedance-derived nutrition parameters on clinical outcomes.

### 3.1. Patients on Peritoneal Dialysis

An earlier study including 48 prevalent African American PD patients showed that a phase angle measured by SF-BIA (BIA-101, RJL/Akern Systems, Clinton Township, MI, USA) was strongly associated with prealbumin and albumin [62]. Similarly, the LTI measured by BIS (Body Composition Monitor, Fresenius Kabi, Bad Homberg, Germany) correlated significantly with albumin, the normalized protein catabolic rate, and handgrip strength (HGS) [49,63].

Quantification of muscle mass and muscle strength are crucial in the diagnosis of sarcopenia. Current guidelines support the measurement of appendicular skeletal mass by BIA [64]. The prevalence of muscle wasting ranged from 19.2% to 40.7% in PD patients depending on the cut-off and the bioimpedance device used [49,57,63]. A recent meta-analysis consisting of 41 studies with 7576 dialysis patients estimated the prevalence of sarcopenia (a reduction in both muscle mass and strength) to be 25.6%, with substantial heterogeneity [65]. Moreover, sarcopenia was seemingly more common in a HD than PD population (26.8% vs. 17.5%, P for interaction = 0.037) [65]. Sarcopenia is the hallmark of frailty, which is increasingly recognized in dialysis patients and associated with hospitalization and death [66]. Among approximately 200 prevalent Chinese PD patients, the LTM by BIS was inversely correlated with a validated Frailty Score (a higher score indicates greater frailty); furthermore, the worsening of frailty tended to aggravate abnormal body composition [67,68]. The bioimpedance technique may help to identify patients in a pre-frail stage and allow for timely intervention. In addition, visceral fat levels determined by MF-BIA were associated with the surrogates of cardiovascular diseases (pulse wave velocity and brachial artery flow-mediated dilation) in PD patients [69].

The complex change in body composition in dialysis patients can be objectively captured by the bioimpedance technique without the need to expose patients to radiation, as is the case for DEXA. An international multicenter observational study (the Initiative for Patient Outcomes in Dialysis-Peritoneal Dialysis [IPOD-PD]) examined the trajectory of body composition by BIS every 3 months in 1054 patients after the beginning of PD [70]. After a minimum follow-up of 3 years, the investigators observed a significant increase in the BMI and FTI, which mainly took place in the first two years, while the LTI remained similar before a decline in the third year. Age, duration of PD, and use of hypertonics were associated with a decrease in the LTI but an increase in the FTI [70]. Another retrospective study of 206 patients on automated peritoneal dialysis (APD) showed that the LTI decreased by a mean of 1.1 kg/m^2^, whereas the FTI increased by 1.9 kg/m^2^ on average over 2 years [71]. But these changes were not predicted by baseline demographics, peritoneal glucose load, or transporter status. Consequently, BIS may be a sensitive tool for monitoring body composition, as a simple increase in the BMI may mask the presence of sarcopenia. This was particularly relevant because the FTI was constantly shown to inversely correlate with the LTI [57,63,71]. Their association was further complicated by the fact that volume overload could lead to a decline in adiposity over one year in a small longitudinal study on Chinese PD patients [57].

An IPOD-PD study revealed a U-shaped association of the BMI with a subdistribution hazard ratio (HR) for mortality [70]. An FTI above the median (11.3 kg/m^2^) was significantly associated with an increase in the risk of death, whereas the opposite was true for an LTI exceeding the median (13.0 kg/m^2^). This highlighted the advantage of bioimpedance, which was able to differentiate an increase in the LTI from the FTI, which clearly conferred a different prognosis. However, neither of them was shown to predict a transfer to HD [70]. In another retrospective cohort of 824 British APD patients, a higher LTI (but not FTI) was significantly associated with improved patient survival (HR 0.86, 95% CI 0.79–0.93) after adjusting for demographics and serum albumin [71]. Interestingly, Kim et al. performed a repeated BIS (Body Composition Monitor, Fresenius Medical Care, Bad Homburg, Germany) in 131 Korean PD patients after a median of 2.4 years [63]. They showed that LTI loss (>10% from baseline) and FTI gain (>10% from baseline) increased the mortality by fourfold after adjusting for comorbidities and nutrition [63]. The association with the FTI, however, was abolished after adjusting for high-sensitivity C-reactive proteins (hs-CRP), while that of LTI remained significant. Nonetheless, this suggested a serial monitoring of body composition may provide additional prognostic information.

### 3.2. Patients on Hemodialysis

The precision of the BMI in determining the nutrition status of HD patients varies with different combinations of LTI and FTI values. By retrieving the body composition data analyzed by BIS among 37,345 HD patients in 17 European countries, Marcelli et al. reported a high prevalence of patients (46.9%) with a reduction in muscle mass (an LTI that was <10th percentile of the reference population) despite a mean BMI of 26.0 kg/m^2^ [72]. For patients with an FTI that was <10th percentile, their BMI ranged from 19.0 ± 1.9 kg/m^2^ to 23.9 ± 3.2 kg/m^2^ across the spectrum of LTI (from <10th percentile to >90th percentile) [72]. Taken together, these results showed that most patients with a low LTI or FTI had normal (or even high) BMI levels according to the World Health Organization, thus suggesting the use of BMI alone in HD patients in the diagnosis of malnutrition/PEW may be misleading.

The correlations of bioimpedance parameters, anthropometrics, and nutrition indexes were examined in 40 prevalent HD patients [73]. The LTI was positively correlated with serum albumin (r = 0.37, *p* = 0.02) and serum prealbumin (r = 0.53, *p* < 0.001), while the FTI was positively correlated with the BMI (r = 0.59, *p* < 0.001), arm circumference (r = 0.44, *p* = 0.004), and triceps skinfold thickness (r = 0.61, *p* < 0.001). Similar to PD patients, the LTM was positively correlated with serum albumin, protein intake, and HGS, and was negatively associated with interleukin-6 [74,75]. In a cross-sectional study of 173 Chinese HD patients, BIA-derived 50 kHz PA was significantly associated with nutrition markers including albumin and the mid-arm circumference [76]. PA was also found to be a stronger predictor of PEW (by ISRNM criteria) than the BMI (odd ratio 4.48, *p* < 0.05), and the optimal phase angle cut-off value to predict PEW was 4.6° (sensitivity: 86.4%, specificity: 76.3%) [76]. Moreover, a 2-year prospective study of 250 maintenance HD patients reported that every 1° increase in PA reduced the risk of hospitalization and cardiovascular events by 21% and 30%, respectively [77].

At present, the determination of appendicular skeletal mass is mainly based on BIA. Lin et al. developed and validated a new equation using BIS parameters to calculate appendicular skeletal muscle mass in HD patients, while taking DEXA as a reference [78]. The equation was evaluated as a diagnostic tool for sarcopenia using cut-offs defined by the Asian Working Group of Sarcopenia, with positive and negative predictive values of 84.2% and 99.6%, respectively. Similar to the PD patients, frailty was not uncommon in HD patients. Among 638 American prevalent HD patients, 30% of them met the definition of frailty based by Fried’s phenotype [79]. Higher FM (odds ratio 1.18, 95% CI 1.02–1.37), lower ICW (odds ratio 0.80, 95% CI 0.73–0.87), but not BMI were associated with higher odds of frailty [79]. BIS parameters were also associated with the individual components of frailty, which offered an opportunity for intervention to modify body composition with an aim to mitigate frailty. Interestingly, Tian et al. examined cognitive functions with the Mini-Mental State Examination Score (MMSE) among 3356 HD patients. Patients with a lower lean-to-fat tissue ratio (<1.27) based on BIS had a significantly higher risk of new onset cognitive impairment (defined as MMSE <27) (HR 1.51, 95% CI 1.24–1.81, *p* < 0.001) [80].

A large multicenter study of 8227 incident HD patients reported that their BMI increased by an average of 0.6 kg/m^2^ over 24 months, which was associated with an increase by 0.95 kg/m^2^ in the FTI and a decrease by 0.4 kg/m^2^ in the LTI, respectively [81]. Registry data suggested that the female gender, diabetes, and a low baseline FTI were associated with a significant greater increase in the FTI, whereas old age, diabetes, the male gender, and a low baseline FTI were associated with a greater decline of LTI [81].

The obesity paradox was well described in HD patients, in which a higher BMI was found to improve survival [82]. Caetano et al. conducted a multicenter observational study in 697 Portuguese HD patients and found that a low FTI was associated with increased mortality (HR 3.25, 95% CI 1.33–7.96) after adjusting for albumin and volume status [83]. Their findings were in line with a recent prospective cohort study on 375 HD patients, which revealed that a higher body fat mass was also associated with a lower mortality (HR 0.95, 95% CI 0.91–1.00) [84]. However, the aforementioned multicenter study with >30,000 HD patients by Marcelli et al. reported that mortality was lowest when both the LTI and FTI were in the 10th to 90th percentile of the corresponding age- and sex-matched healthy populations [72]. The mortality was increased with a low LTI (<10th percentile) and at the two extremes of the FTI [72]. Importantly, patients having both an LTI and FTI that were in <10th percentile had the highest mortality (HR 2.51, 95% CI 2.12–2.96, *p* < 0.001). Since patients with an LTI at <10th percentile and an FTI at >90th percentile had a relatively lower mortality (HR 1.74, 95% CI 1.4–2.17, *p* < 0.001), the authors postulated that a high FTI apparently only exerts a protective effect when the LTI is low. Likewise, two retrospective European cohorts using BIS to assess body composition showed that a low LTI (<10th percentile) significantly increases the risk of mortality by approximately 60% [85,86]. Recently, there has been growing interest in using the ratio of ECW to ICW (ECW/ICW) as a composite index of fluid overload and malnutrition. Yajima et al. showed that the ECW/ICW (when measured by MF-BIA) was negatively associated with a simplified creatinine index (a surrogate for muscle mass) in 224 prevalent HD patients [87]. Moreover, both ECW/ICW (≥0.57) and s simplified creatinine index (<20.4 g/kg/day) were independent predictors of all-cause mortality, and this is after the adjustment of demographics, comorbidities, and albumin levels. One of the drawbacks of this composite index is that an elevated ECW/ICW can be attributed by either fluid overload or malnutrition, or both. Therefore, targeted intervention for abnormal body compartment may not be easy.

**Figure 1:**
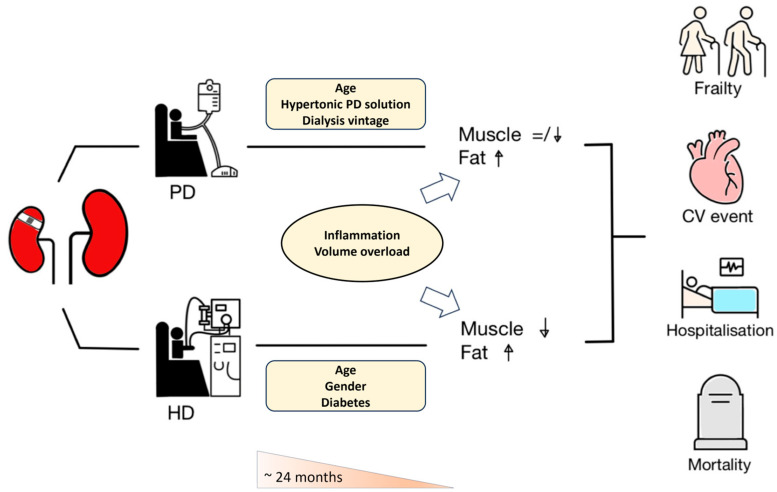
The evolution of body composition in dialysis patients was affected by both patient-specific and treatment-specific factors. Abbreviations: CV, cardiovascular; HD, hemodialysis; and PD, peritoneal dialysis.

Figure 1 illustrates the evolution of body compositions after an initiation of dialysis and their possible consequences on clinical outcomes. It is important to note that abnormal changes in muscle mass or fat mass often co-exist with other adverse prognostic factors, most commonly fluid overload and inflammation. In a cohort of 8883 European HD patients, a low LTI was seldom observed as an isolated phenomenon [88]. Instead, 40% of patients in this cohort had a low LTI in combination with fluid overload and/or inflammation. Fluid overload was most severe in patients with a depletion of both ATM and LTM, as well as concomitant inflammation (defined as a CRP of >6 mg/L). Furthermore, patients with a combination of low LTI, fluid overload, and inflammation had the highest risk of death (HR 5.89, 95% CI 2.28–8.01) [88]. This coupling of muscle wasting, malnutrition, and inflammation was similarly observed in an IPOD-PD study [70]. Interestingly, persistent fluid overload may deplete fat stores through the natriuretic peptide-induced browning of white adipose tissue [89]; salt accumulation in tissue may also cause muscle degradation by inducing hypercatabolism at the cellular level [90]. Also, the OH was also associated with both the severity of the frailty on a continuous scale [67] and individual domains of the frailty phenotype [79]. Collectively, we propose a new term, ‘FIFA complex’, to characterize the intricate link between Frailty, Inflammation, Fluid overload, and Atherosclerotic disease in ESKD patients [44].

## 4. Clinical Implications of Bioimpedance Technique: Toward an Integrated Nutritional Assessment in Dialysis Patients

It is important to recognize that no single nutrition marker is perfect. The current KDOQI guidelines suggest nutrition screening to be performed at least biannually in dialysis patients, but it acknowledges that there was a lack of evidence to make recommendations on the most optimal screening tool [56]. Commonly used screening tools, such as the Malnutrition Universal Screening Tool, the Malnutrition Screening Tool, and the Geriatric Nutrition Risk Index, primarily rely on the assessment of BMI or changes in body weight, which may not be sensitive enough to detect changes in separate body compartments in patients with ESKD; furthermore, each tool showed inconsistent magnitudes of correlation with SGA and MIS [56]. Conversely, none of these screening tools directly measure fat or muscle mass, which constitute the diagnostic domains of PEW. Given the wealth of evidence supporting the prognostic value of bioimpedance-derived nutrition parameters, the bioimpedance technique may serve as a simple bedside nutrition screening tool for ESKD patients. Assessments of different body compartments can usually be completed within 15 to 30 min when patient is waiting outside the consultation room. The early detection of PEW is crucial for timely intervention. However, changes in body weight during the early course of dialysis may cause confusion to clinicians [8]. For example, an increase in body weight could be attributed to improvements in nutrition status secondary to the correction of uremia and increases in dietary intake, while it could be equally possible due to fluid accumulation that occurs owing to inappropriate dry weights shortly after dialysis. The 3C model of BIS may be particularly useful to disentangle early between fluid overload, adiposity, and muscle wasting. In a small cohort of 91 HD patients, insufficient nutritional status was reflected by a BIS decision tree (LTI <10th percentile, FTI <10th percentile, and ECW > 15%) that independently predicted the development of PEW, which provided an opportunity of early detection and intervention [91]. Nevertheless, it should be noted that the reference value of body composition parameters in the Body Composition Monitor (Fresenius Medical Care, Bad Homburg, Germany) (the most commonly employed BIS device from the literature) were derived from Caucasians [27]. A recent report established the reference ranges of LTI and FTI from 1305 Asians [92], but it is unlikely that these values have been incorporated into existing devices, which implies that the clinicians need to proactively take reference to it during nutrition assessment.

It Is, however, important to recognize that the measurement of body composition only constitutes part of the nutritional assessment of dialysis patients, and it should be complemented by assessments of dietary intake, anthropometric parameters, laboratory biomarkers (albumin, prealbumin, CRP, phosphate, vitamin D level, and the parathyroid hormone (PTH)) [56]. Emerging evidence has suggested mineral bone disease in CKD patients plays a more direct role in the development of PEW, which may be mediated by altered muscle and fat metabolism [93]. Specifically, elevated fibroblast growth factor-23, which is frequently induced by hyperphosphatemia, can be directly stimulated via the hepatic production of inflammatory cytokines [94]. A pre-clinical study showed that the PTH/PTH receptor pathway may be responsible for cachexia through adipose browning and muscle atrophy [95]. In addition, recent guidelines have supported the diagnosis of sarcopenia, which shares similar pathological risk factors to PEW and should be based on the combined assessments of muscle mass, muscle strength, and physical performance [64]. Although muscle mass is one of the key determining factors for muscle strength, studies have suggested a stronger association between muscle strength and functional performance, which contributes to frailty [96,97,98]. Muscle strength can be readily measured by handheld dynamometers and expressed in HGS, while a functional status can be assessed by 6 m gait speed or a timed up-to-go test [64]. Although low serum albumin is the hallmark of PEW, clinicians should also be cautious about the confounding effect of hypervolemia or inflammation. Taken together, we believe that bioimpedance should be considered as part of the comprehensive nutritional assessment of PD patients alongside SGA, muscle strength, and physical performance [14]. In addition, bioimpedance technique may have an additional advantage in the serial monitoring of nutrition status owing to the absence of radiation exposure. Furthermore, SGA might be more capable of detecting changes in symptoms, whilst bioimpedance provides objective assessments of body composition even in the absence of symptoms.

It is important to recognize that sarcopenia and PEW are two distinct but overlapping syndromes. Sarcopenia is characterized by reduction in muscle mass (which can be measured by BIA or DEXA) and muscle strength. The prevalence in dialysis patients was estimated to be 25.6% (95% CI 22.1 to 29.4%) by a recent meta-analysis [65]. On the other hand, PEW represents a state of depleted body protein (muscle) and energy stores, which is driven by inadequate nutrient intake and hypercatabolism [6]. The prevalence in dialysis patient showed a wide range from 28 to 54% [7]. These two conditions are commonly associated with inflammation, and they are phenotypically manifested as frailty.

## 5. Conclusions and Future Directions

The bioimpedance technique enables simple, convenient, and highly reproducible assessments of volume and nutrition in ESKD patients at bedside. Despite its widespread use and advantages, some clinicians still doubt the validity and accuracy of the bioimpedance technique in ESKD patients. Suffice to say, the published evidence suggests good agreement between bioimpedance and reference methods at the population level, but individual variability does exist. However, the susceptibility of bias from hydration status and disagreements with other reference methods have made the role of DEXA as the gold standard questionable. Interestingly, assessments of the LTM and ATM by BIS did not appear to be biased by the hydration markers derived by echocardiographic parameters. Further validation studies may not be rewarding as there is a certain degree of inherent error when conducting bioimpedance, whereas it might be more meaningful if future studies are able to define the clinically acceptable limits of accuracy.

The bioimpedance technique has the potential to be used as a nutrition screening tool that can detect subclinical volume overload and sarcopenic obesity, both of which could occur in the early stage of dialysis and are insufficiently addressed by BMI. The 3C model of BIS may be particularly useful as it segregates a whole body into the OH, LTM, and ATM. Moreover, serial bioimpedance measurements could provide objective assessments of the evolution of body compositions. More importantly, the bioimpedance technique should not be taken as a single nutritional assessment tool but as part of an integrated and comprehensive assessment, which also encompasses muscle strength, physical performance, and health-related quality of life. To achieve these goals, the development and implementation of standardized measurement protocols are of paramount importance.

However, clinical trials that have examined the added benefits of incorporating the bioimpedance technique into routine care are lacking. Counterintuitively, recent randomized controlled trials that applied bioimpedance-guided volume management did not result in significant benefits in the clinical endpoints (including all-cause mortality and residual renal function) [99,100,101]. We believe future research in the following areas may help to bridge the existing the knowledge gap:

- What is the optimal cut-off of bioimpedance-derived parameters (e.g., LTI or FTI) to identify or diagnose malnutrition, as well as in predicting the PEW (or other complications) in patients with ESKD?
- Defining the clinically acceptable limit of accuracy for the bioimpedance technique.
- Does modification of bioimpedance-derived parameters by nutrition intervention result in improvements in clinical endpoints?
- How frequently should bioimpedance be performed in ESKD patients to screen and monitor nutrition status?

## Figures and Tables

**Table 1:** Comparisons of the different types of bioimpedance techniques.

	Single-Frequency BIA	Multi-Frequency BIA	Bioimpedance Spectroscopy
Frequency of current	Single frequency at 50 kHz	Multiple fixed frequencies (commonly at 1, 5, 50, 250, 500, and 1000 kHz)	A spectrum of frequencies (at least 50 frequencies from 5 to 1000 kHz)
Physiological model	Two-compartment model (FM and FFM)	Two-compartment model (FM and FFM)	Three-compartment model (OH, ATM, and LTM)
Mathematical algorithm	Bioimpedance data fit into linear regression equation (derived from specific reference population)	Bioimpedance data fit into linear regression equation (derived from specific reference population)	Bioimpedance data fit into the Cole model (nonlinear least-square curve fitting model) to calculate the volume of body compartments, the result of which are applied to the three-compartment model by Chamney et al. [25]
Output parameters	Phase angle, edema index, and vector analysis	Phase angle and edema index	OH, LTI, and FTI
Examples of devices	BIA 450 (Biodynamics^®^, Seattle, WA, USA)	Inbody 720 (Biospace, Seoul, Republic of Korea)	Body Composition Monitor (Fresenius Medical Care, Bad Homburg, Germany)

Abbreviations: ATM, adipose tissue mass; BIA, bioelectrical impedance analysis; FFM, fat-free mass; FM, fat mass; FTI, fat tissue index; LTI, lean tissue index; LTM, lean tissue mass; and OH, overhydration.

**Table 2:** BIA-derived equations for the prediction of different body compartments.

**Author, Year**	**Subjects**	**BIA Device**	**Reference Method**	**Equation**	**R^2^**	**SEE**
Kyle et al., 2004 [29]	343 White healthy subjects aged 2–94 years	SF-BIA (Xitron 4000B; ImpediMed, Carlsbad, CA, USA)	DEXA (Hologic QDR-4500; Hologic, Bedford, MA, USA)	FFM (kg) = −4.104 + (0.518 × height^2^/resistance) + (0.231 × weight) + (0.130 × reactance) + (4.229 × sex: men = 1, women = 0)	0.97	1.72 kg
Sun et al., 2003 [30]	1474 White and 355 Black subjects aged 12–94 years	SF-BIA (model 101; RJL Systems, Inc., Detroit, MI, USA)	TBW: deuterium dilution FFM: DEXA (Lunar Inc., Madison, WI, USA)	Male: TBW (L) = 1.20 + 0.45 × height^2^/resistance + 0.18 × weight Female: TBW (L) = 3.75 + 0.45 × height^2^/resistance + 0.11 × weight Male: FFM (kg) = −10.68 + 0.65 × height^2^/resistance + 0.26 × weight + 0.02 × resistance Female: FFM (kg) = −9.53 + 0.69 × height^2^/resistance + 0.17 × weight + 0.02 × resistance	0.84 0.79 0.90 0.83	3.8 L 2.6 L 3.9 kg 2.9 kg
Dey et al., 2003 [31]	101 Swedish elderly subjects (≥70 years)	SF-BIA (model 101; RJL Systems, Inc., Detroit, MI, USA)	Four compartment models	FFM (kg) = 11.78 + (0.499 × height^2^/resistance) + (0.134 × weight) + (3.449 × Sex) FM (kg) = weight − FFM	0.95	2.64 kg
Deurenberg et al., 1995 [32]	137 Dutch healthy controls	MF-BIA (Dietosytem, Milano, Italy)	TBW: deuterium dilution ECW: bromide dilution	TBW (L) = 6.69 + (0.35 × height^2^/resistance [at 100 kHz]) + (0.17 × weight) − (0.11 × age) + (2.66 × sex: men = 1, women = 0) ECW (L) = 2.30 + (0.20 x height^2^/resistance [at 1 kHz]) + (0.07 × weight) – (0.02 × age)	0.95 0.89	1.73 L 0.98 L
Barbosa-Silva et al., 2005 [33]	1967 healthy controls (multiethnicity)	SF-BIA (model 101; RJL Systems, Mt Clemens, MI, USA)	N/A	Phase angle (degree) = arc tangent ratio of reactance to resistance × (180/π)	0.49	N/A

It should be noted that the prediction equations are population-specific. Clinicians are suggested to choose the one that is derived from the population with characteristics that closely match those of the study subjects. It was suggested that the prediction error (SEE) of 2.0–2.5 kg in men and 1.5–1.8 kg in women is acceptable [23]. Abbreviations: BIA, bioelectrical impedance analysis; DEXA, dual X-ray absorptiometry; FFM, fat-free mass; FM, fat mass; MF-BIA, multi-frequency bioelectrical impedance analysis; R^2^, correlation coefficient; SEE, standard error of estimate; and SF-BIA, single-frequency bioelectrical impedance analysis.

**Table 3:** Proposed elements to be included in a standardized protocol for bioimpedance measurement.

Domains	Comments	Remarks
Instrument related		
Device	Consistent signal of reproducible amplitude	Regular calibration Same machine is preferred in serial measurements
Electrodes	Electrodes should be placed according to the manufacturers’ instructions and should not be reused	Two electrodes on the dorsum of a hand (one on the head of the metacarpal and one on the mid-point between the styloid processes of radius and ulnar) and foot (one on the head of the metatarsal and one on the mid-point between medial and lateral malleoli), respectively (preferably on the same side in subsequent measurements). The proximity (<5 cm) of electrodes should be avoided
Subject related		
Position	Supine with each limb slightly away from the body (30–45 degrees)	Standing is associated with a transient decrease in impedance
Skin temperature	Non-febrile subjects in ambient temperatures	Cutaneous vasodilation lowers impedance
Food and drinks	Fasting for at least 4 h is preferred	Consumption of food and beverages may decrease impedance by 4–15 ohms
Exercise	Avoid exercise for 8 h	Exercise approximately reduces resistance by 3% and reactance by 8% immediately after exercise
Environment	Avoid touching the metallic frame of a bed	Electrical interference
Disease related		
Chronic kidney disease	Ideally measured in the euvolemic state (especially for SF-BIA and MF-BIA)	The determination of lean mass may be confounded by hypervolemia (see detailed discussion in Section 2.3)
Peritoneal dialysis	Ideally performed with an ‘empty abdomen’ (i.e., peritoneal dialysis solution drained out)	The absolute difference of parameters between a ‘full’ and ‘empty’ abdomen is small with uncertain clinical significance (see detailed discussion in Section 2.2)
Hemodialysis	Measurements should be performed 60 min after hemodialysis Do not place the electrodes on the side of the body with an arteriovenous dialysis fistula or when the central venous catheter is connected to a dialysis machine	Lean mass decreases and fat mass increases after hemodialysis, and these changes correlate with the changes of extracellular water removed during dialysis

Abbreviations: MF-BIA, multi-frequency bioelectrical impedance analysis; SF-BIA, single-frequency bioelectrical impedance analysis.

## Data Availability

Not applicable.

## Supplementary Materials

The following supporting information can be downloaded at: https://www.mdpi.com/article/10.3390/nu16010015/s1, Table S1. Reference range of the body compartments determined by different BIA-derived equations. References [29,30,31,32,33] are cited in supplementary file.

## Abbreviations

ATM, adipose tissue mass; BIA, bioelectrical impedance analysis; BIS, bioimpedance spectroscopy; BMI, body mass index; CKD, chronic kidney disease; CRP; C-reactive protein; DEXA, dual X-ray absorptiometry; ECW, extracellular water; ESKD, end-stage kidney disease; FFM, fat-free mass; FM, fat mass; FTI, fat tissue index; ICW, intracellular water; HD; hemodialysis; HGS, handgrip strength; LTI, lean tissue index; LTM, lean tissue mass; MF-BIA, multi-frequency bioelectrical impedance analysis; MIS, malnutrition–inflammation score; OH, volume of overhydration; PA, phase angle; PD; peritoneal dialysis; PEW, protein-energy wasting; PTH, parathyroid hormone; SF-BIA, single-frequency bioelectrical impedance analysis; SGA, subjective global assessment; and TBW, total body water.
