# Supplementary material for: Nutritional Assessments by Bioimpedance Technique in Dialysis Patients
Source: Nutrients. 2023 Dec 20;16(1):15. doi: 10.3390/nu16010015 (PMC10780416; doi:10.3390/nu16010015)
Supplement: Supplementary file 1 [file nutrients-16-00015-s001.zip › nutrients-2705195-supplementary.pdf]

Table S1. Reference range of body compartments determined by different BIA-derived equations

Author, year	Subjects	Reference range by BIA	Remarks
Kyle et al, 2004 [1]	343 White healthy subjects aged 2-94 years	Male FFM (kg):<br>20-29y: 61.7 ± 5.9<br>30-39y: 63.2 ± 6.2<br>40-49y: 63.1 ± 3.9<br>50-59y: 63.3 ± 6.2<br>60-69y: 59.2 ± 6.1<br>70-79y: 56.7 ± 5.2<br>>80y: 54.0 ± 6.0<br><br>Women FFM (kg):<br>20-29y: 44.9 ± 2.6<br>30-39y: 45.1 ± 3.6<br>40-49y: 44.8 ± 5.6<br>50-59y: 45.8 ± 6.5<br>60-69y: 43.0 ± 4.5<br>70-79y: 42.2 ± 5.2<br>>80y: 38.8 ± 3.1	
Sun et al, 2003 [2]	1474 Whites and 355 Blacks aged 12–94 years	Not specified	For both TBW and FFM, equation tended to underestimate in Black and overestimate in White
Dey et al, 2003 [3]	101 Swedish elderly (≥70 years)	FFM (kg):<br>70y: 50.4 ± 9<br>75y: 48.7 ± 8<br><br>FM (kg)<br>70y: 25.5 ± 8.3<br>75y: 22.3 ± 7.2	Reference values calculated by applying equation to 2 elderly cohorts (500 aged 70y and 323 aged 75 y)

Deurenberg et al, 1995 [4]	137 Dutch healthy controls	Not specified	Prediction of ECW and TBW is dependent on body water distribution
Barbosa-Silva et al, 2005 [5]	1967 healthy controls (multiethnicity)	Male phase angle (degree):<br>18-20y: 7.90 ± 0.47<br>20-39y: 8.02 ± 0.75<br>30-39y: 8.01 ± 0.85<br>40-49y: 7.76 ± 0.85<br>50-59y: 7.31 ± 0.89<br>60-69y: 6.96 ± 1.10<br>≥ 70y: 6.19 ± 0.97<br><br>Female phase angle (degree):<br>18-20y: 7.04 ± 0.85<br>20-39y: 6.98 ± 0.92<br>30-39y: 6.87 ± 0.84<br>40-49y: 6.91 ± 0.85<br>50-59y: 6.55 ± 0.87<br>60-69y: 5.97 ± 0.83<br>≥ 70y: 5.64 ± 1.02	Mean of phase angle was 7.48 ± 1.10° for men, and 6.53 ± 1.01° for women respectively.<br>Phase angle was significantly lower in women than in men and was inversely correlated with age

References:

1. Kyle UG, Genton L, Karsegard L, Slosman DO, Pichard C. Single prediction equation for bioelectrical impedance analysis in adults aged 20--94 years. Nutrition. 2001 Mar;17(3):248-53.
2. Sun SS, Chumlea WC, Heymsfield SB, Lukaski HC, Schoeller D, Friedl K, Kuczmarski RJ, Flegal KM, Johnson CL, Hubbard VS. Development of bioelectrical impedance analysis prediction equations for body composition with the use of a multicomponent model for use in epidemiologic surveys. Am J Clin Nutr. 2003 Feb;77(2):331-40.
3. Dey, D., Bosaeus, I., Lissner, L. et al. Body composition estimated by bioelectrical impedance in the Swedish elderly. Development of population-based prediction equation and reference values of fat-free mass and body fat for 70- and 75-y olds. Eur J Clin Nutr. 2003;57, 909–916.

4. Deurenberg P, Tagliabue A, Schouten FJM. Multi-frequency impedance for the prediction of extracellular water and total body water. British Journal of Nutrition. 1995;73(3):349-358.
5. Barbosa-Silva MC, Barros AJ, Wang J, Heymsfield SB, Pierson RN Jr. Bioelectrical impedance analysis: population reference values for phase angle by age and sex. Am J Clin Nutr. 2005 Jul;82(1):49-52.
